# Zinc ferrite nanoparticle-induced cytotoxicity and oxidative stress in different human cells

**DOI:** 10.1186/s13578-015-0046-6

**Published:** 2015-09-17

**Authors:** Hisham A. Alhadlaq, Mohd Javed Akhtar, Maqusood Ahamed

**Affiliations:** Department of Physics and Astronomy, College of Science, King Saud University, Riyadh, 11451 Saudi Arabia; King Abdullah Institute for Nanotechnology, King Saud University, Riyadh, 11451 Saudi Arabia

**Keywords:** Zinc ferrite nanoparticles, A549, A431, HepG2, Human health, Cytotoxicity, Reactive oxygen species

## Abstract

**Background:**

Zinc ferrite nanoparticles (NPs) have shown potential to be used in biomedical field such as magnetic resonance imaging and hyperthermia. However, there is limited information concerning the biological response of zinc ferrite NPs. This study was designed to evaluate the cytotoxicity of zinc ferrite NPs in three widely used in vitro cell culture models: human lung epithelial (A549), skin epithelial (A431) and liver (HepG2) cells. Zinc ferrite NPs were characterized by electron microscopy and dynamic light scattering. Cell viability, cell membrane damage, reactive oxygen species (ROS), glutathione (GSH), mitochondrial membrane potential (MMP), transcriptional level of apoptotic genes were determined in zinc ferrite NPs exposed cells.

**Results:**

Zinc ferrite NPs were almost spherical shaped with an average size of 44 nm. Zinc ferrite NPs induced dose-dependent cytotoxicity (MTT and LDH) and oxidative stress (ROS and GSH) in all three types of cells in the dosage range of 10–40 µg/ml. Transcriptional level of tumor suppressor gene p53 and apoptotic genes (bax, caspase-3 and caspase-9) were up-regulated while the anti-apoptotic gene bcl-2 was down-regulated in cells after zinc ferrite NPs exposure. Furthermore, higher activity of caspase-3 and caspase-9 enzymes was also observed in zinc ferrite NPs treated cells. ROS generation, MMP loss and cell death in all three types of cells were abrogated by *N*-acetyl cysteine (ROS scavenger), which suggests that oxidative stress might be one of the plausible mechanisms of zinc ferrite NPs cytotoxicity. It is worth mentioning that there was marginally difference in the sensitivity of three cell lines against zinc ferrite NPs exposure. Cytotoxicity of zinc ferrite NPs were in following order; A549 > HepG2 > A431.

**Conclusion:**

Altogether, zinc ferrite NPs induced cytotoxicity and oxidative stress in A549, A431 and HepG2 cells, which is likely to be mediated through ROS generation. This study warrants further investigation to explore the potential mechanisms of toxicity of zinc ferrite NPs in normal cells as well as in animal models.

## Background

Magnetic nanoparticles (NPs) hold great promise in biomedical applications including hyperthermia, biosensor, magnetic resonance imaging (MRI) and drug delivery [1–3]. Over the years, magnetite (Fe_3_O_4_) was the most studied magnetic NPs. In the last decade, it became easier to develop new and more effective types of magnetic NPs [4]. Spinel ferrite NPs with the general formula MFe_2_O_4_ (where M is A is divalent cation of Zn, Ni, Mn, Mg or Co) are very important materials because of their interesting magnetic and electrical properties with good chemical and thermal stabilities [5]. However, their biomedical application may ultimately be limited, because of the limited knowledge on biological response of these NPs [6, 7]. Therefore, it is necessary to explore the biological fate and possible toxic response of magnetic NPs for their successful implications in biomedical field.

Zinc ferrite (ZnFe_2_O_4_) NPs, a lesser investigated class of spinel ferrites, frequently utilized as a contrasting agent in MRI and spintronics devices [8]. Despite the potential application of zinc ferrite NPs, there is a serious lack of information concerning the toxicity of this material both at in vitro and in vivo level. Some significant studies demonstrated the toxic effects of zinc ferrite NPs. Recently, Saquib et al. [9] suggested that zinc ferrite NPs trigger apoptosis and/or necrosis in human amnion epithelial (WISH) cells through mitochondria dependent intrinsic apoptotic pathway. Zinc ferrite NPs have also been reported to induce chromosomal aberration in the meristematic root cells of sunflower [10].

Toxicological investigations using animal models are both expensive and time taking. Therefore, a search for suitable alternative in vitro models to precisely predict the in vivo toxicity is indispensable. Human cell lines have been shown to be good in vitro models and sensitive tools for high-throughput toxicity screening and have the potential to reduce the use of animals in toxicity studies. Cell lines can be utilized for the generation of mechanistic data that could potentially be used for toxicity assessments. Therefore, we have chosen three widely used in vitro cell culture models; human lung epithelial (A549), skin epithelial (A431) and liver (HepG2) cells as tools to explore the cytotoxicity of zinc ferrite NPs.

Exposure to NPs could occur through the air, skin, food and the use of medical devices [11]. The respiratory tract and skin represents the main route of NPs exposure for workers employed in the manufacturing and handling. The NPs have high possibility to deposit in the respiratory system and can retain in the lungs for a long time where they provoke oxidative stress and an inflammatory burden with respect to their fine-sized equivalents [12, 13]. On the other hand, liver is a primary site of NPs accumulation after they get entry to circulatory system [14]. Studies suggest that NPs get absorbed as they pass through the gastrointestinal tract and distributed different vital organs including liver via the circulatory system [15, 16]. Therefore, we have selected human lung (A549), skin (A431) and liver (HepG2) cell lines to explore cytotoxic response of zinc ferrite NPs. These cell lines have been widely used in nanotoxicity research [17–20].

The underlying mechanisms of toxicity of NPs are not fully explored. One mechanism more often argued is the induction of oxidative damage of cell macromolecules, either due to the reactive oxygen species (ROS) generation or by inactivation of antioxidant defense system [21, 22]. ROS generation is a crucial factor not only in apoptotic pathway, but also in genetic damage, inflammation and several other cellular processes [23, 24]. Our earlier studies have shown that magnetic NPs have potential to induce ROS mediated cytotoxicity in different human cells [20, 25, 26]. In the present study, we investigated the cytotoxicity and oxidative stress response of zinc ferrite NPs in three different types of human cells (A549, A431 and HepG2). To achieve this goal, we determined the cell viability, cell membrane damage, ROS, glutathione (GSH), mitochondrial membrane potential (MMP) and transcriptional level of some apoptotic genes in zinc ferrite NPs exposed cells.

## Methods

### Chemicals and reagents

Dulbecco’s modified eagle’s medium (DMEM), penicillin–streptomycin and fetal bovine serum were purchased from Invitrogen (Carlsbad, CA, USA). The 3-(4,5-dimethylthiazol-2-yl)-2, 5-diphenyltetrazoliumbromide (MTT), 3-amino-7-dimethylamino-2-methyl-phenazine hydrochloride (neutral red), *N*-acetylcysteine (NAC), rhodamine-123 (Rh123) and 2,7-dichlorofluorescin diacetate (DCFH-DA) were bought from Sigma-Aldrich (St. Louis, MO, USA). Kits for lactate dehydrogenase (LDH) and caspase assays were obtained from Bio-Vision Inc. (Milpitas, CA, USA). Other chemicals were of analytical grade and bought from available commercial sources.

### Zinc ferrite nanoparticles

Dry nanopowder of zinc ferrite (ZnFe_2_O_4_) [Product No. 633844, <100 nm particle size (BET), >99 % trace metals basis] was obtained from Sigma-Aldrich (St. Louis, MO, USA). Zinc ferrite NPs were further characterized in our laboratory.

### Zinc ferrite nanoparticle characterization

Structural characterization of zinc ferrite NPs was done by field emission scanning electron microscope (FESEM, JSM-7600F, JEOL Inc., Japan) and field emission transmission electron microscopy (FETEM, JEM-2100F, JEOL Inc., Japan) at an accelerating voltage of 15 and 200 kV, respectively. In FETEM study, 1 mg/ml suspension of zinc ferrite NPs was prepared in de-ionized water. Then suspension was sonicated at room temperature for 15 min at 40 W to avoid NPs agglomeration. To determine the size of NPs, stock suspension was diluted to appropriate working solutions. Further, a drop of aqueous suspension of zinc ferrite NPs was poured onto a carbon-coated copper grid, air-dried and FETEM measurements were performed.

Behavior of zinc ferrite NPs in aqueous state (e.g. water and culture medium) was evaluated by dynamic light scattering (DLS) (Nano-ZetaSizer-HT, Malvern, UK) as reported elsewhere [27]. Briefly, zinc ferrite nano-powder were suspended in de-ionized water and culture medium at the concentration of 40 μg/ml. This suspension was further sonicated at room temperature for 15 min at 40 W and performed the DLS measurements. We have utilized concentration of 40 µg/ml for DLS study because this was the highest dosage level employed in cytotoxicity studies.

### Cell culture and exposure of zinc ferrite nanoparticles

A549, A431 and HepG2 cells were obtained from American Type Culture Collection (ATCC) (Manassas, VA, USA). Cells were cultured in DMEM medium supplemented with 10 % FBS and 100 U/ml penicillin–streptomycin at 5 % CO_2_ and 37 °C. At 85 % confluence, cells were harvested using 0.25 % trypsin and were sub-cultured for further experiments. Cells were allowed to attach on the surface of culture flask for 24 h before NP exposure. Dry powder of zinc ferrite NPs was suspended in DMEM medium at a concentration of 1 mg/ml and diluted to appropriate dosages (10–40 µg/ml). The different concentrations of NPs were then sonicated at room temperature for 15 min at 40 W to avoid agglomeration of NPs before exposure to cell. In some experiments, cells were pre-exposed for 1 h with 10 mM of NAC before 24 h co-exposure with or without zinc ferrite NPs. Cells not exposed to zinc ferrite NPs served as controls in each experiment. Selection of 10–40 µg/ml dosage range of zinc ferrite NPs was based on preliminary dose–response experiments (data not shown).

### Cell viability assay

Cell viability assay was done following the method as described by Mossman [28] with few changes [25]. This assay evaluates the mitochondrial function by determining the ability of living cells to reduce MTT into blue formazon product. Briefly, 10,000 cells/well were seeded in 96-well plates and exposed to different concentrations of zinc ferrite NPs (10–40 µg/ml) for 24 h. After the treatment time completed, culture medium was taken out from each well to avoid interference of NPs and replaced with new medium containing MTT solution in an amount equal to 10 % of culture volume and incubated for 3 h at 37 °C until a purple-colored formazan product developed. The resulting formazan product was dissolved in acidified isopropanol. Then, 96-well plate was centrifuged at 2300*g* for 5 min to settle down the remaining NPs. Further, 100 µl supernatant was transferred to new 96-well plate, and the absorbance was taken at 570 nm utilizing a microplate reader (Synergy-HT, BioTek, USA).

### Lactate dehydrogenase leakage assay

Lactate dehydrogenase (LDH) assay was carried out using a BioVision LDH-cytotoxicity colorimetric assay kit as per the manufacturer’s instruction. Briefly, 10,000 cells/well were seeded in 96-well plates and treated to different concentrations of zinc ferrite NPs (10–40 µg/ml) for 24 h. At the end of the exposure time, 96-well plate was centrifuged at 2300*g* for 5 min to settle down the NPs. Then, 100 μl of the supernatant was transferred to a new 96-well plate that already contained 100 μl of the reaction mixture from the BioVision kit and incubated for 30 min at room temperature. After the incubation time completed, absorbance of the solution was determined at 340 nm with help of a microplate reader (Synergy-HT, BioTek, USA). The LDH levels in the culture medium versus those present within cells were measured and compared with the control values according to the manufacturer’s protocol.

### Reactive oxygen species assay

Intracellular reactive oxygen species (ROS) generation after the treatment of zinc ferrite NPs was evaluated using 2,7-dichlorofluorescin diacetate (DCFH-DA) as reported by Wang and Joseph [29] with few changes described in our previous publication [30]. The ROS level was measured by two methods; fluorometric quantitative assay by micro-plate reader and cell imaging by fluorescence microscopy. For fluorometric assay, 10,000 cells/well were seeded in 96-well black-bottomed culture plates and allowed to attach on the surface for 24 h in a CO_2_ incubator at 37 °C. Further, cells were treated with zinc ferrite NPs (10–40 µg/ml) for 24 h. After the exposure completed, were washed twice with HBSS before being incubated in 1 ml of working solution of DCFH-DA for 30 min at 37 °C. After this, cells were lysed in alkaline solution and centrifuged at 2300*g* for 10 min. A 200 μl supernatant was transferred to a new 96-well plate, and fluorescence was measured at 485 nm excitation and 520 nm emission utilizing the microplate reader (Synergy-HT, BioTek, USA). The values were presented as a percent of fluorescence intensity relative to the controls.

A parallel set of cells (5 × 10^4^ cells/well in a 24-well transparent plate) were assayed for intracellular fluorescence using a fluorescence microscope (OLYMPUS CKX 41), with images captured at the magnification of 20×.

### Cell extract preparation

Crude cell extract were prepared according to the protocol described in our earlier work [31]. Cell extract were used for glutathione (GSH), caspase-3 and caapase-9 enzymes assays. In brief, cells were cultured in 75-cm^2^ culture flask and treated with zinc ferrite NPs (10–80 µg/ml) for 24 h. After the exposure completed, cells were harvested in ice-cold phosphate buffer saline by scraping and washed with phosphate buffer saline at 4 °C. The cell pellets were then lysed in cell lysis buffer [1 × 20 mM Tris–HCl (pH 7.5), 150 mM NaCl, 1 mM Na_2_EDTA, 1 % Triton, 2.5 mM sodium pyrophosphate]. Following centrifugation (15,000*g* for 10 min at 4 °C) the cell extract (supernatant) was stored in ice for biochemical assays.

### Glutathione assay

Intracellular glutathione (GSH) content was estimated utilizing Ellman’s method [32]. In brief, a mixture of 0.1 ml of crude cell extract and 0.9 ml of 5 % TCA was centrifuged at 2300*g* for 15 min. After that 0.5 ml of the supernatant was added into 1.5 ml of 0.01 % DTNB and the reaction was monitored at 412 nm. The content of GSH was presented in terms of nmole/mg protein.

### Mitochondrial membrane potential assay

Mitochondrial membrane potential (MMP) was estimated using the method of Zhang et al. [33] with few changes [34]. Briefly, cells (5 × 10^4^ cells/well) were exposed to different concentrations (10–40 µg/ml) of zinc ferrite NPs for 24 h. After the completion of exposure time, cells were harvested and washed twice with PBS. Then, cells were treated with 10 µg/ml of Rh-123 fluorescent dye for 1 h at 37 °C in dark. Furthermore, cells were washed twice with PBS then the fluorescence intensity of Rh-123 dye was determined using upright fluorescence microscope (OLYMPUS CKX 41) by grabbing the images at 20× magnification.

A parallel set of cells (1 × 10^4^ cells/well) in 96-well plate were analyzed for quantification of Rh-123 using the microplate reader (Synergy-HT, BioTek, USA).

### Quantitative assay of apoptotic genes by real-time PCR

Cells were cultured in 6-well plates and treated with zinc ferrite NPs at a dosage of 20 μg/ml for 24 h. After the completion of exposure time, total RNA was extracted by Qiagen RNeasy mini Kit (Valencia, CA, USA) according to the manufacturer’s protocol. The RNA content was estimated using Nanodrop 8000 spectrophotometer (Thermo-Scientific, Wilmington, DE, USA), and the integrity of RNA was visualized on a 1 % agarose gel using the gel documentation system (Universal Hood II, BioRad, Hercules, CA, USA). The first strand of cDNA was synthesized from 1 μg of total RNA by the reverse transcriptase using M-MLV (Promega, Madison, WI, USA) and oligo (dT) primers (Promega) according to the manufacturer’s instructions. Quantitative real-time PCR was performed by QuantiTect SYBR Green PCR kit (Qiagen) using the ABI PRISM 7900HT Sequence Detection System (Applied Biosystems, Foster City, CA, USA). Two microliters of template cDNA was added to the final volume of 20 μl of reaction mixture. Real-time PCR cycle parameters included 10 min at 95 °C followed by 40 cycles involving denaturation at 95 °C for 15 s, annealing at 60 °C for 20 s, and elongation at 72 °C for 20 s. The sequences of the specific sets of primer for p53, bax, bcl-2, caspase-3, caspase-9 and β-actin utilized in the present investigation are given in our previous study [25]. Expressions of selected genes were normalized to the β-actin gene, which was used as controls.

### Determination of caspase-3 and caspase-9 enzymes activity

Caspase-3 and caspase-9 enzymes activity was evaluated in exposed and control cells using Bio-Vision colorimetric assay kits. Preparation of cell extract is reported above. This assay is based on the principle that activated caspases in apoptotic cells cleave the synthetic substrates to release free chromophore *p*-nitroanilide (pNA), which is measured at 405 nm. The pNA was generated after specific action of caspase-3 and caspase-9 on tertrapeptide substrates DEVD-pNA and LEHD-pNA, respectively [25, 35]. In brief, reaction mixture consisted of 50 µl of cell extract protein (as prepared above), 50 µl of 2× reaction buffer (containing 10 mM dithiothreitol) and 5 µl of 4 mM DEVD-pNA (for caspase-3) or LEHD-pNA (for caspase-9) substrate in a total volume of 105 µl. The reaction mixture was incubated at 37 °C for 1 h and absorbance of the final product was estimated utilizing the microplate reader (Synergy-HT, BioTek, USA) at 405 nm according to kit’s instruction.

### Protein assay

Protein content was estimated by the Bradford method [36] using bovine serum albumin as the standard.

### Statistical analysis

Statistical significance was measured by one-way analysis of variance followed by Dunnett’s multiple comparison tests. Significance was ascribed at p < 0.05.

## Results

### Zinc ferrite nanoparticle characterization

**Fig. 1:**
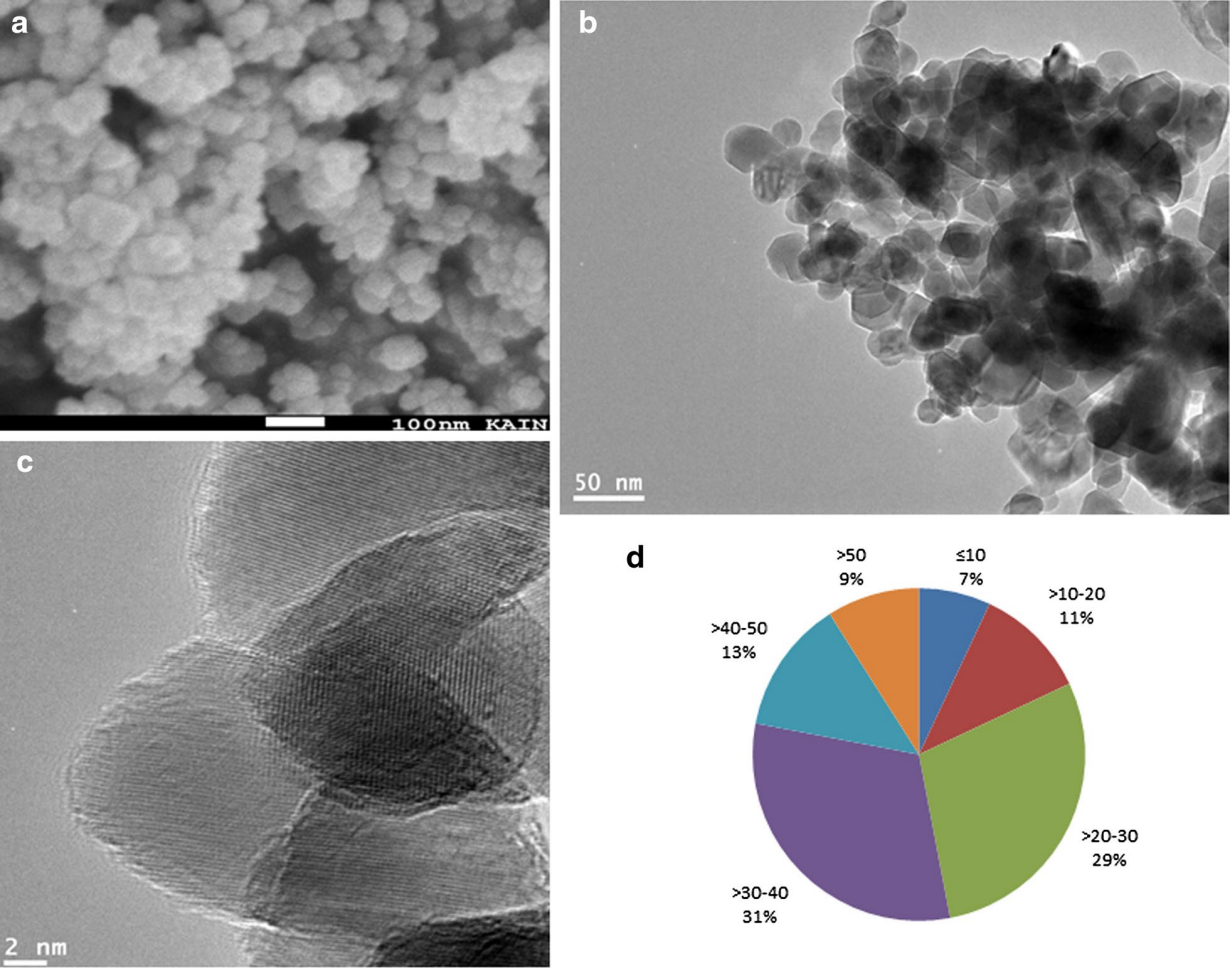
Electron microscopy characterization of zinc ferrite NPs. **a** FESEM image, **b** FETEM image, **c** FETEM image with high resolution and **d** frequency of particle size distribution

Techniques such as FETEM, FESEM and DLS was used to characterize the zinc ferrite NPs. FESEM image depicts the surface morphology zinc ferrite NPs (Fig. 1a). Figure 1b shows the typical TEM image of zinc ferrite NPs. As we can see in Fig. 1c the high resolution TEM indicated the crystalline behavior of zinc ferrite NPs. TEM and SEM images have shown that particles were almost spherical with smooth surfaces. The average size of NPs was calculated from estimating over 120 particles in random fields of TEM view. The average size of zinc NPs was approximately 44.3 nm. Figure 1d shows the size distribution frequency of zinc ferrite NPs.

Characterization of zinc ferrite NPs in aqueous state by DLS technique is given in Table 1. Secondary particle size (hydrodynamic size) of zinc ferrite NPs in de-ionized water and cell culture medium was 312 and 289 nm, respectively. Further, zeta-potential of zinc ferrite NPs in water and culture medium was −18 and −23 mV, respectively.

**Table 1:** Dynamic light scattering characterization of zinc ferrite NPs

	De-ionized water (mean ± SD)	Culture media (mean ± SD)
DLS characterization of zinc ferrite NPs		
Hydrodynamic size (nm)	312 ± 67	289 ± 55
Zeta potential (−mV)	18 ± 8	23 ± 7

### Zinc ferrite nanoparticles reduced the cell viability

**Fig. 2:**
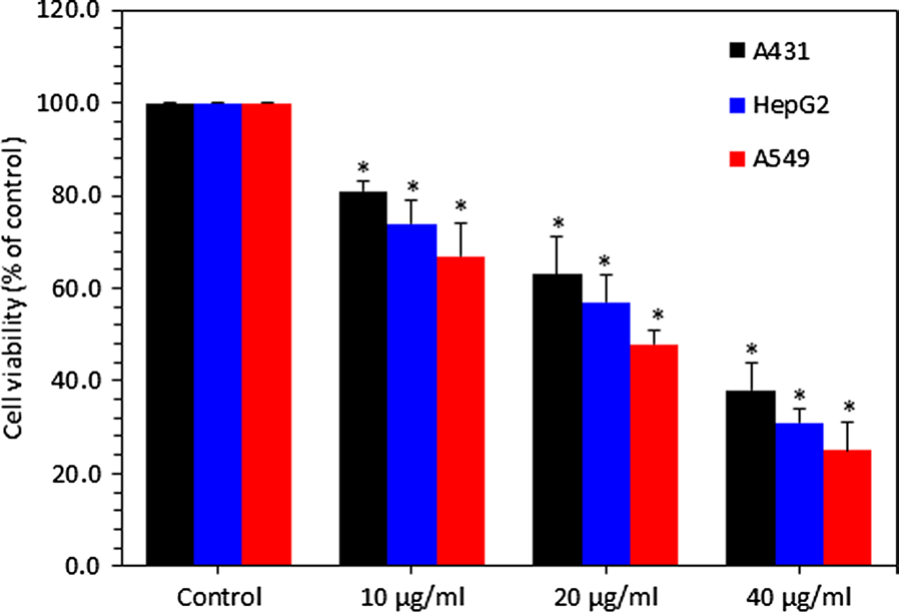
Zinc ferrite NPs reduced the viability of A431, HepG2 and A549 cells. Data represented are mean ± SD of three identical experiments made in three replicate. *Significant difference as compared to the controls (p < 0.05 for each)

A431, A549 and HepG2 cells were treated with zinc ferrite NPs (10–40 µg/ml) for 24 h and cell viability was determined by MTT assay. Results have shown that zinc ferrite NPs significantly decreased the viability of all three types of cells in a dose-dependent manner. We also observed that zinc ferrite NPs induced cytotoxicity in following order A549 cells > HepG2 cells > A431 cells. Cell viability was decreased to 81, 63 and 38 % for A431 and 74, 57 and 31 % for HepG2, while 67, 48 and 25 % for A549 cells, at the concentrations of 10, 20, and 40 µg/ml, respectively (p < 0.05 for each) (Fig. 2).

### Zinc ferrite nanoparticle-induced membrane damage

**Fig. 3:**
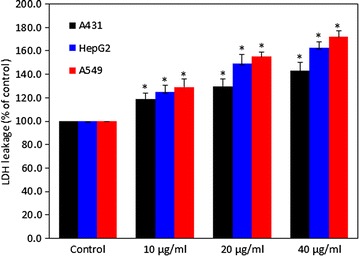
Zinc ferrite NPs induced membrane damage in A431, HepG2 and A549 cells. Data represented are mean ± SD of three identical experiments made in three replicate. *Significant difference as compared to the controls (p < 0.05 for each)

LDH is an enzyme widely found in the cytosol that converts lactate to pyruvate. When the integrity of plasma membrane is disrupted, LDH leaks into media and its extracellular content increases depending upon the extent of NPs cytotoxicity. Therefore, higher level of LDH in culture medium indicates higher cytotoxic response of NPs. We also observed that zinc ferrite NPs induced LDH leakage dose-dependently in all three types of cells (Fig. 3). Effects of zinc ferrite NPs on A549 cells was higher than those of HepG2 and A431 cells. LDH leakage in A431 cells was increased to 119, 130 and 143 %, and 125, 149 and 163 % for HepG2, while 129, 155 and 172 % were A549 cells for the dosages of 10, 20 and 40 µg/ml, respectively (p < 0.05 for each) (Fig. 3).

### Zinc ferrite nanoparticle-induced reactive oxygen species generation

**Fig. 4:**
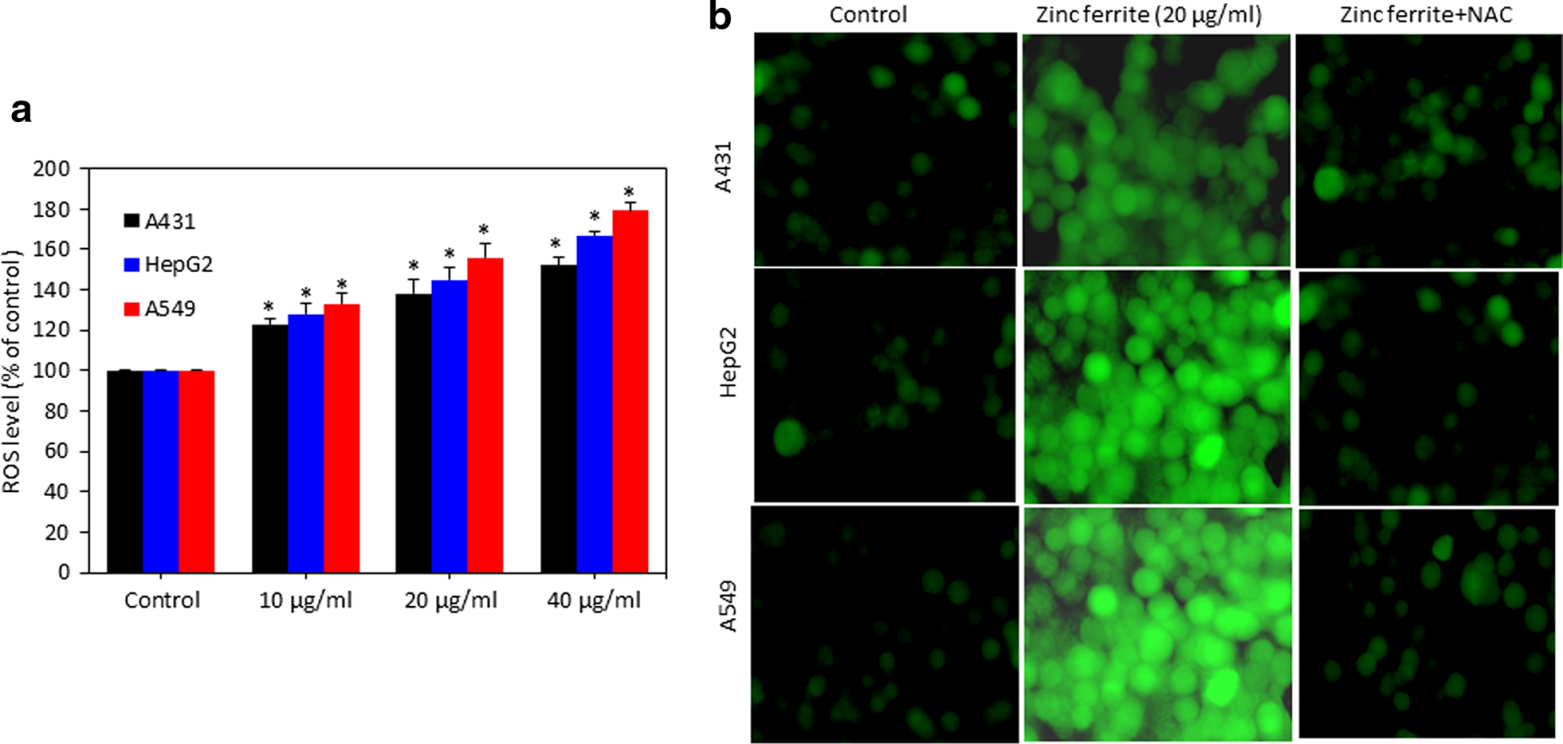
Zinc ferrite NPs induced ROS generation in A431, HepG2 and A549 cells. **a** Percentage change in ROS generation in all three cells after zinc ferrite NPs exposure at the concentrations of 0, 10, 20 and 40 µg/ml for 24 h. **b** Representative microphotographs showing ROS generation in A431, HepG2 and A549 cells after zinc ferrite NPs exposure at a concentration of 20 µg/ml for 24 h. Images were captured with a fluorescence microscope (OLYMPUS CKX 41). Data represented are mean ± SD of three identical experiments made in three replicate. *Significant difference as compared to the controls (p < 0.05 for each)

ROS generation has been suggested to be involved in several cellular events including inflammation, senescence mutation, DNA damage and apoptosis. We evaluated the potential of zinc NPs to induce intracellular ROS generation in A431, HepG2 and A549 cells. Quantitative results indicated that zinc ferrite NPs induced ROS generation dose-dependently in all three types of cells (p < 0.05 for each) (Fig. 4a). Fluorescent microscopy results also suggested that zinc ferrite NPs treated cells show higher intensity of green fluoresce of DCF dye (ROS generation marker) than those of the control cells (Fig. 4b).

### Zinc ferrite nanoparticle-reduced glutathione level

**Fig. 5:**
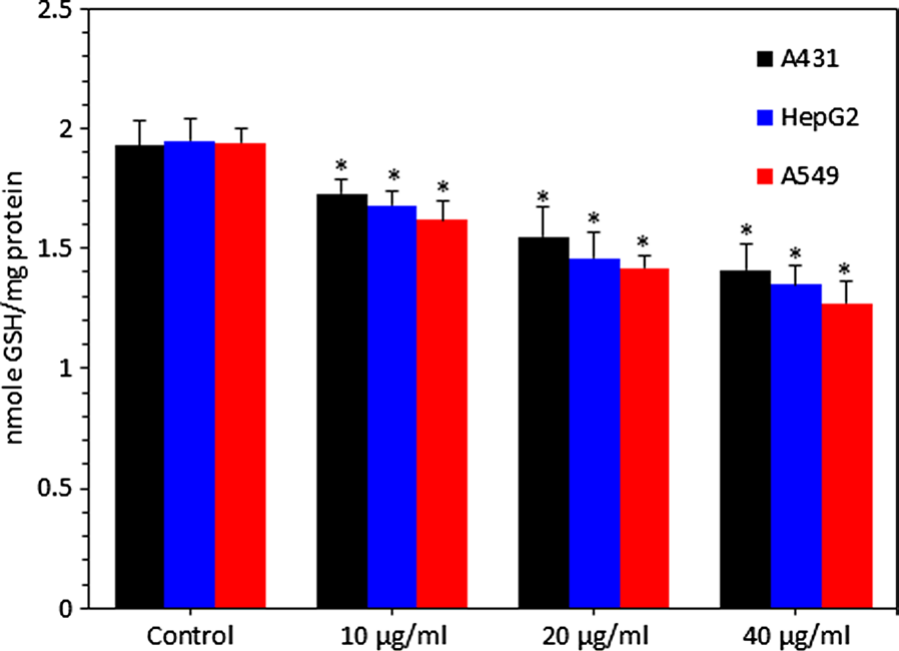
Zinc ferrite NPs induced GSH depletion in A431, HepG2 and A549 cells. Data represented are mean ± SD of three identical experiments made in three replicate. *Significant difference as compared to the controls (p < 0.05 for each)

Antioxidant GSH depletion and changes in the activity of various antioxidant enzymes indicative of lipid peroxidation have been implicated in oxidative damage of cellular macromolecules [37]. Therefore, we further examined the GSH level in A431, HepG2 and A549 cells exposed to zinc ferrite NPs at the concentrations of 10, 20 and 40 µg/ml for 24 h. Results showed that zinc ferrite NPs reduced the level of GSH in all three types of cell in a dose-dependent manner (Fig. 5).

### Zinc ferrite nanoparticle-reduced mitochondrial membrane potential

**Fig. 6:**
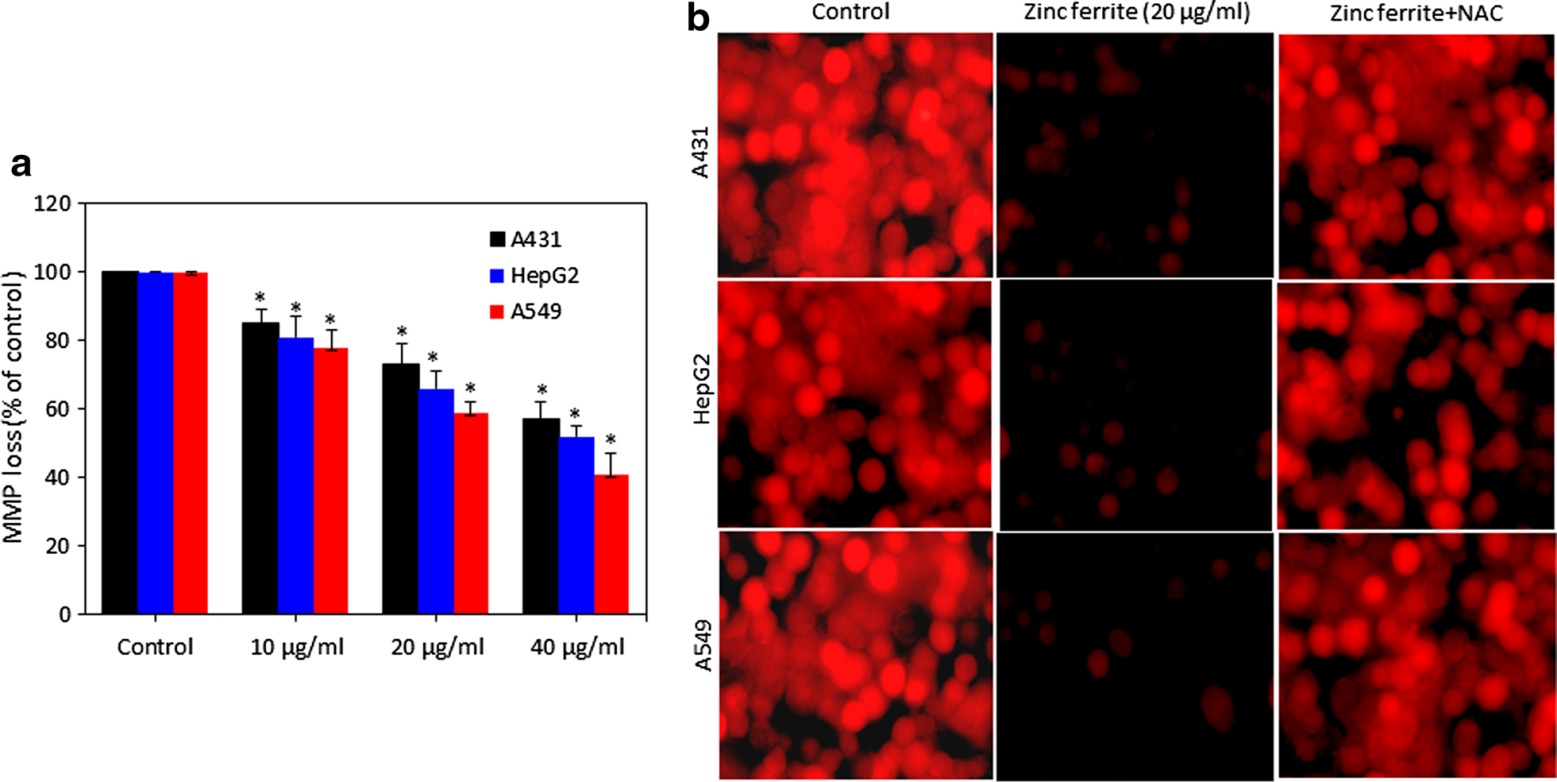
Zinc ferrite NPs induced MMP loss in A431, HepG2 and A549 cells. **a** Percentage change in MMP in all three cells after zinc ferrite NPs exposure at the concentrations of 0, 10, 20 and 40 µg/ml for 24 h. **b** Representative microphotographs showing MMP loss in A431, HepG2 and A549 cells after zinc ferrite NPs exposure at a concentration of 20 µg/ml for 24 h. Images were captured with a fluorescence microscope (OLYMPUS CKX 41). Data represented are mean ± SD of three identical experiments made in three replicate. *Statistically significant difference as compared to control (p < 0.05)

Earlier studies have shown that MMP loss during the process of apoptosis [23]. We studied the effect of zinc ferrite NPs on MMP in A431, HepG2 and A549 cells. Quantitative data showed that zinc ferrite NPs induced MMP loss in a dose-dependent manner in all three types of cells (p < 0.05 for each) (Fig. 6a). Fluorescence microscopy results were also according to the quantitative data. The brightness of the fluorescent intensity was decreased in cells treated with zinc ferrite NPs that shows a significant loss MMP (Fig. 6b).

### Zinc ferrite nanoparticle-altered the expression of apoptotic genes

**Fig. 7:**
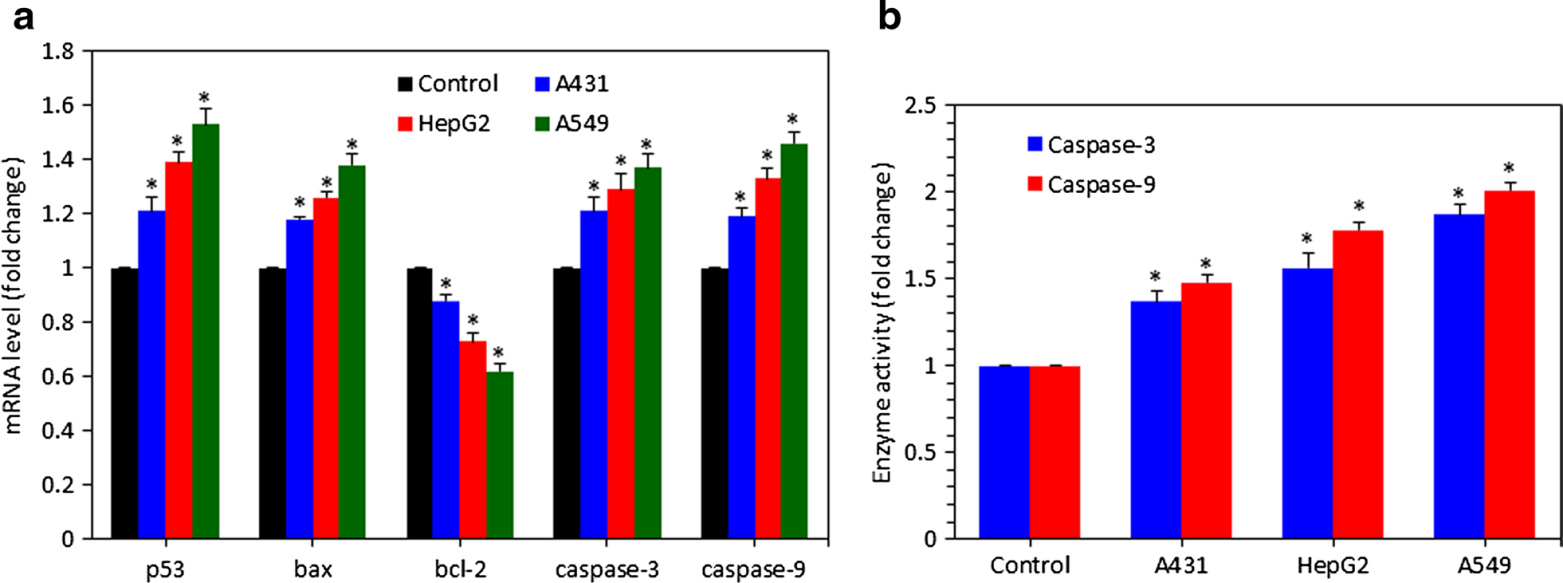
Zinc ferrite NPs induced apoptotic response in A431, HepG2 and A549 cells. **a** Regulation of mRNA levels of apoptotic genes in all the cells after exposure to zinc ferrite NPs at a concentration of 20 µg/ml for 24 h. The mRNA levels were determined by quantitative real-time PCR as described in “Methods” section. **b** Activity of caspase-3 and caspase-9 enzymes in A431, HepG2 and A549 cells after exposure to zinc ferrite NPs at a concentration of 20 µg/ml for 24 h. Data represented are mean ± SD of three identical experiments made in three replicate. *Significant difference as compared to the controls (p < 0.05 for each)

Quantitative real-time PCR was used to analyze the expression of mRNA level of various genes involved in apoptosis (p53, bax, bcl-2, caspase-3 and caspase-9) in A431, HepG2 and A549 cells exposed to zinc ferrite NPs at a concentration of 20 µg/ml for 24 h. We noticed that zinc ferrite NPs altered the expression of these genes in all three types of cells (p < 0.05 for each) (Fig. 7a). Transcription expression level of tumor suppressor gene p53 and pro-apoptotic gene bax were up-regulated while the expression of anti-apoptotic gene bcl-2 was down-regulated in cells treated with zinc ferrite NPs. We also observed the higher expression of caspase-3 and caspase-9 genes in NPs treated cells as compared to the control.

### Zinc ferrite nanoparticle-induced the activity of caspase-3 and caspase-9 enzymes

In support of real-time PCR results, we further determined the activity of caspase-3 and caspase-9 enzymes in A431, HepG2 and A549 cells treated with zinc ferrite NPs at a concentration of 20 µg/ml for 24 h. Results have shown that zinc ferrite NPs increased the activity of both apoptotic enzymes (caspas-3 and caspase-9) in all three types of cells (p < 0.05 for each) (Fig. 7b).

### Zinc ferrite nanoparticle- induced cytotoxicity through ROS generation

**Fig. 8:**
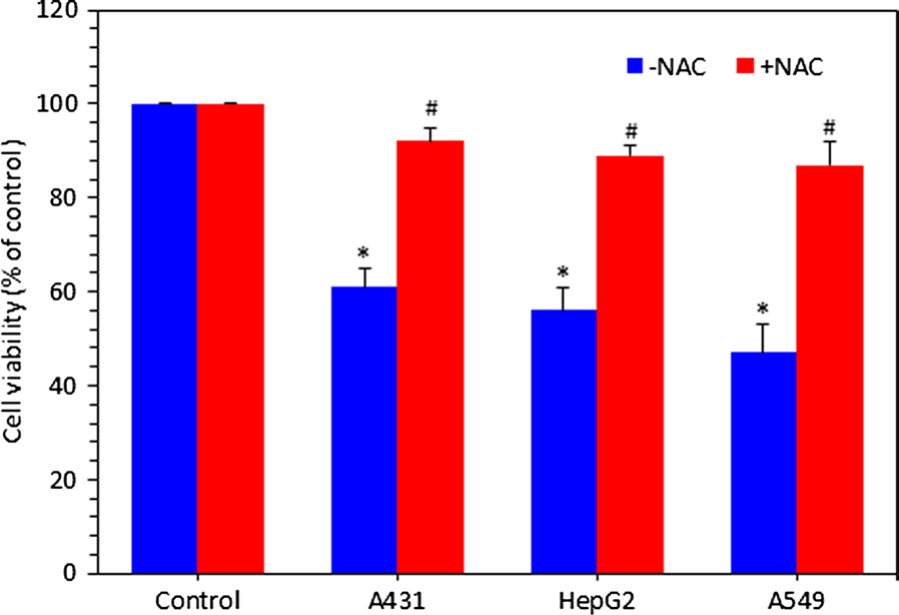
Zinc ferrite NPs induced cytotoxicity in A431, HepG2 and A549 cells through ROS generation. Cells were exposed to zinc ferrite NPs at a concentration of 20 µg/ml in the presence of 10 mM *N*-acetyl-cystein (NAC) for 24 h. At the end of the exposure time, cell viability was determined. We observed that NAC significantly preserved the viability of all three cells induced by zinc ferrite NPs. Data represented are mean ± SD of three identical experiments made in three replicate. *Significant difference as compared to the controls (p < 0.05 for each). ^#^Significant inhibitory effect of NAC on cell viability reduction (p < 0.05 for each)

In order to investigate whether ROS generation might plays a critical role in cytotoxicity of zinc ferrite NPs, cells were exposed to zinc ferrite NPs in the presence of NAC (ROS scavenger). Results showed that NAC abolished almost fully the cytotoxic effect of zinc ferrite NPs in all three types of cells (A431, HepG2 and A549) (Fig. 8).

Taken together, there was marginally difference in the sensitivity of three cells against zinc ferrite NPs exposure. Cytotoxic response of zinc ferrite NPs were in following order; A549 cells > HepG2 cells > A431cells. However, the modes of action of cytotoxicity of zinc ferrite NPs in all three cells were look similar.

## Discussion

Despite many advantages in using spinel ferrite NPs, evidences are increasing to indicate that spinet ferrite NPs may cause toxic effects to human cells [9, 20, 25, 38]. Although the beneficial effects of zinc ferrite (ZnFe_2_O_4)_ have attracted considerable attention in biomedical application, their human and environmental hazards should also be considered. Therefore, in this study, we have examined the toxicological effects of zinc ferrite NPs in three different cell lines of human body. The NPs may pose toxic effects due to their tiny size and unique physiochemical properties [27]. Therefore, it is essential to characterize the NPs before their toxicological investigations. The principal parameters of NPs are their size, shape, crystallanity, purity/impurity, hydrodynamic size and agglomeration/dispersion that influence the biological interaction of NPs [21, 27, 39]. We have employed SEM, TEM and DLS techniques to characterize the NPs. The SEM image suggested the smooth surfaces of NPs. The primary particle size of zinc ferrite measured by TEM was approximately 44 nm. High-resolution TEM images represented the crystalline behavior of this material. Secondary particle size (aqueous suspension) of the NPs is also regarded as an important parameter for in vitro assays. Therefore, extent of aggregation and secondary size of zinc ferrite NPs in water and cell culture medium was evaluated through dynamic light scattering (DLS). The DLS is widely used to determine the size of Brownian NPs in colloidal suspensions in the nano and submicron ranges [44]. We observed that the secondary particle size (hydrodynamic diameter) of zinc ferrite NPs was several times higher as compared with the primary size measured by TEM. The greater size of NPs in liquid medium than the primary size could be due to tendency of NPs to aggregate in aqueous state. This finding is supported by other studies, [40, 41] and has been briefly discussed in our earlier work [42]. The average hydrodynamic size of NPs in cell culture medium were slightly smaller (289 nm) as compared to de-ionized water (312 nm), which indicates the possible interaction of zinc ferrite NPs with the protein of culture media, which has been widely reported with different NPs that leads to the formation of ‘protein corona’ [43, 44]. The tendency of particles to form aggregates depends on the surface modification and charge. The surface charge of zinc ferrite NPs measured as zeta potential was around −18 and −23 mV for water and culture medium, respectively.

After characterization of zinc ferrite NPs, we performed a series of tests to determine the cytotoxic potential of zinc ferrite NPs in in vitro systems. Because of the limited metabolic capacity of the in vitro models, the biotransformation of a chemical in vitro may be minimal compared with that in in vivo systems. However, despite their known limited metabolic capacity, in vitro cell lines still represent promising tools for the development of high-throughput, predictive and mechanism-based assays to evaluate the potential toxicity of agents such as NPs [26]. As demonstrated in Fig. 2, it is evident that A549, A431 and HepG2 cells responded differently to zinc ferrite NPs exposure. The viability values indicate that A549 cells were more sensitive to the NPs exposure than A431 and HepG2 cells after 24 h. Decrease in cell viability after zinc ferrite NPs treatment were in following order; A549 cells > HepG2 cells > A431 cells. LDH leakage from cells is also an evidence of cell membrane damage. Studies have shown that LDH level was elevated in cells culture medium after exposure of cells to the magnetic NPs [20, 45]. Our results also demonstrated that LDH leakage was higher in a dose-dependent manner in all three types of cells treated with zinc ferrite NPs.

Normally, NPs enter into cells through endocytosis and localize in the vacuoles and cell cytoplasm of the cells [46, 47]. During internalization by endocytosis, magnetic NPs may undergo dissolution or degradation due to acidic pH of endosomes, [48] and release iron (Fe) ions that most likely promotes the generation of ROS via Haber–Weiss and/or Fenton reactions, and consequently cause oxidative damage to cell macromolecules [49]. Fe ions can also potentially escape into the cytoplasm and become a part of accessible Fe ions called the labile iron pool, which has also been shown to exist in the nucleus [50, 51]. Moreover, the tiny size with large surface area makes NPs more compatible for generation of ROS in the cell. Cancer cells as compared to normal cells are under greater intrinsic oxidative stress due to alterations in metabolism and higher accumulation of ROS due to an imbalance between ROS generation and elimination [52]. Higher sensitivity of cancer cells toward ROS generation due to NPs exposure as compared to normal cells makes them more suitable to explore the potential mechanisms of ROS mediated toxicity of NPs. We also observed ROS level was significantly higher whereas the antioxidant GSH level was significantly lower in all three A549, A431 and HepG2 cells exposed to zinc ferrite NPs. Moreover, induction ROS generation as well as reduction in cell viability due to zinc ferrite NPs exposure was efficiently prevented by antioxidant NAC treatment. These findings suggest that ROS generation might be the primarily responsible for the cytotoxicity of zinc ferrite NPs. This observation was according to the earlier findings in various human cell lines that indicate ROS generation and oxidative stress due to magnetic NPs [9, 53].

We further observed MMP loss against zinc ferrite NPs exposure in all three human cells. Changes in mitochondrial activity was based on cationic fluorescent probe Rh123, indicated the role of oxidative stress in toxicity of zinc ferrite NPs. We assume that lesser fluorescence intensity of Rh123 indicates perturbation of inner mitochondrial membranes, and consequently mitochondrial dysfunction as a result of ROS. Because ROS scavenger NAC significantly prevents the loss of MMP in zinc ferrite NPs treated cells. The transcriptional data on modulation of p53 and bax/bcl-2 ratio and release of caspases have strengthened the role of zinc ferrite in inducing mitochondrial dependent apoptotic pathway. In general, the caspases are crucial for the activation and execution of apoptosis. The main intrinsic pathway is characterized by mitochondrial dysfunction, with the release of cytochrome c, activation of caspase 9, and subsequently of caspase 3 enzyme [54]. Typically, p53 is activated when DNA damage occurs or cells are stressed and p53 is translocated to the nucleus, where it can induce pro-apoptotic gene expression on the mitochondrial membrane and activate the effector caspases and accelerate cell death [9, 55].

## Conclusion

Our results have shown that zinc ferrite NPs induced cytotoxicity (MTT and LDH) in human lung (A549), skin (A431) and (HepG2) cells in a dose-dependent manner. Zinc ferrite NPs were also found to induce oxidative stress dose-dependently, indicated by ROS generation and glutathione depletion. Cytotoxicity of zinc ferrite NPs was effectively abolished *N*-acetyl-cysteine (ROS scavenger) indicating that oxidative stress might be one of the possible causes of zinc ferrite NPs toxicity. Zinc ferrite NPs also showed apoptotic response in all three cells evident by loss of MMP and alteration in the regulation of the apoptotic genes (p53, bax, bcl-2, caspase-3, and caspase-9). The sensitivity of these three cells against zinc ferrite NPs exposure was marginally different. Cytotoxic response of zinc ferrite NPs were in following order; A549 cells > HepG2 cells > A431 cells. Overall, present investigation suggests that zinc ferrite NPs induced cytotoxicity was mediated through ROS generation. Further studies are underway to explore the toxic potential of zinc ferrite NPs at in vivo level.
